# Freedom comes at a cost?: An exploratory study on affordances’ impact on users’ perception of a social robot

**DOI:** 10.3389/frobt.2024.1288818

**Published:** 2024-03-18

**Authors:** Guanyu Huang, Roger K. Moore

**Affiliations:** The Speech and Hearing Research Group (SpandH), Department of Computer Science, University of Sheffield, Sheffield, United Kingdom

**Keywords:** human-robot interaction (HRI), affordance, anthropomorphism, spoken interaction, use cases, mixed-method approach

## Abstract

Along with the development of speech and language technologies, the market for speech-enabled human-robot interactions (HRI) has grown in recent years. However, it is found that people feel their conversational interactions with such robots are far from satisfactory. One of the reasons is the habitability gap, where the usability of a speech-enabled agent drops when its flexibility increases. For social robots, such flexibility is reflected in the diverse choice of robots’ appearances, sounds and behaviours, which shape a robot’s ‘affordance’. Whilst designers or users have enjoyed the freedom of constructing a social robot by integrating off-the-shelf technologies, such freedom comes at a potential cost: the users’ perceptions and satisfaction. Designing appropriate affordances is essential for the quality of HRI. It is hypothesised that a social robot with aligned affordances could create an appropriate perception of the robot and increase users’ satisfaction when speaking with it. Given that previous studies of affordance alignment mainly focus on one interface’s characteristics and face-voice match, we aim to deepen our understanding of affordance alignment with a robot’s behaviours and use cases. In particular, we investigate how a robot’s affordances affect users’ perceptions in different types of use cases. For this purpose, we conducted an exploratory experiment that included three different affordance settings (adult-like, child-like, and robot-like) and three use cases (informative, emotional, and hybrid). Participants were invited to talk to social robots in person. A mixed-methods approach was employed for quantitative and qualitative analysis of 156 interaction samples. The results show that static affordance (face and voice) has a statistically significant effect on the perceived warmth of the first impression; use cases affect people’s perceptions more on perceived competence and warmth before and after interactions. In addition, it shows the importance of aligning static affordance with behavioural affordance. General design principles of behavioural affordances are proposed. We anticipate that our empirical evidence will provide a clearer guideline for speech-enabled social robots’ affordance design. It will be a starting point for more sophisticated design guidelines. For example, personalised affordance design for individual or group users in different contexts.

## 1 Introduction

In recent years, people have shown great interest in interacting with speech-enabled artificial agents, such as voice assistants in smartphones or smart speakers and social robots with embodiment. Springing from the ‘command and control systems’ of the 1970s, these agents now can act more actively in social interactions in the healthcare, education, entertainment and other social industries (Bartneck et al., 2020, p.163). Given the diversity of the use cases, there is a need to explore the appropriate designs of social robot interfaces in different use cases. What should a social robot look, sound, and behave like in given situations? The design challenges are several-fold. For example, how to increase an agent’s social acceptability so people feel comfortable interacting with it (Dautenhahn, 2007); how to avoid the phenomenon known as the ‘uncanny valley’ (Mori et al., 2012), where a negative emotional response would be triggered when an artificial agent is very human-like but not convincingly so; also, how to increase discoverability (Norman, 2013, p10) to help users figure out how an agent works and what operations are possible.

In this last regard, we conduct our study within the framework of the affordance theory. Adopted from Gibsonian psychology (Gibson, 1977), affordance in the design field refers to the relationship between a physical object and an interacting agent, which affects people’s perception of a physical object and determines how the object could possibly be used (Norman and Love, 2004; Norman, 2008; 2013). According to this theory, perceivable affordances of a social robot are related to its appearance, sound and behaviours (e.g., facial expressions and language behaviours). Previously, many studies of the social robot’s design focused on the uncanny valley. For example, perceptual mismatches of facial features (MacDorman and Chattopadhyay, 2016) and face-voice stimulus (Mitchell et al., 2011) could reduce users’ sense of affinity. Bearing in mind the importance of aligning face and voice stimulus, our focus in this study is to examine the alignment of a robot’s static and behavioural affordances in various use cases. Specifically, our goal is to examine whether and how a social robot’s affordances should align with its designated roles in different types of use cases. Our research interests are about how a robot’s affordances affect people’s perception of it before and after interactions in different use cases. For example, would people favour a more robot-like agent for an informative use case and a more human-like agent for an emotional use case? If so, how?

To address these questions, we adopted a mixed-method approach to examine statistical relations and gain insights from listening to participants’ voices. The quantitative method explores potential correlative relationships between independent variables (affordances and use cases) and people’s perceptions of the robot. The data is collected via questionnaires before and after a participant interacts with a robot. The qualitative method collects data from semi-structured interviews after the experiment and generates thematic analysis. The social robot ‘Furhat’ is used in the experiment because it can change faces and voices in the same embodiment (Furhat Robotics, 2023). Two independent variables are examined in various settings: the robot’s appearance and voice (adult-like, child-like and robot-like) and use cases (informative, emotional and hybrid). The results show that use cases have statistical significance in affecting people’s first impressions of a robot’s competence, warmth and their motivation to interact with the robot, as well as post-interaction perceived competence and warmth, especially between informative and emotional use cases. Static affordances, such as face and voice, have statistical significance in affecting people’s first impressions of a robot’s warmth but no statistical significance on post-interaction perceptions. In addition, it shows controversial views of aligning static affordance with behavioural affordance. Some mismatches may cause decreased perceptions (e.g., a child-like robot behaves rudely). Some mismatches may be unexpectedly funny (e.g., a robot-like robot says ‘sweet’).

The paper begins by explaining why affordance design matters in the case of social robots. It then examines evidence and challenges of multi-modality in affordance design. It moves on to explain the materials and methods used in the study, with emphasis given to explaining the design of interactive use cases and measurements used to collect data. The findings section first presents the quantitative analysis of the survey results and then identifies the main themes drawn from the qualitative analysis of the semi-structured interview. The general discussion reflects on the main findings, which answer the research questions and limits of the current work. In the conclusion section, the practical impact of this study is highlighted. Opportunities for future work are identified.

## 2 Background and related work

### 2.1 Affordance design of social robots

What should a robot look like, sound like and behave like? One classic principle for robot design is the ‘form-function fit’ (Bartneck et al., 2020). It means that the form of a robot needs to reflect its function. This principle has been well tested and applied for robots which provide labour services, such as floor cleaning robots (Prassler et al., 2000), a feeding robot (Nanavati et al., 2023) and a navigation robot (Asakawa, 2023). In recent decades, robots have been used more widely in social domains, such as education, healthcare, and entertainment (Bartneck et al., 2020, p.163). What a robot provides progresses from visible, tangible physical labour value to more abstract information and emotional value. Social robots go beyond carrying out orders in *Command and Control Systems* and providing information in *Interactive Voice Response Systems*; they can also provide emotional support in social domains (Moore, 2017). The appliance of the ‘form-function fit’ principle faces more dynamic and complicated situations, such as providing socially assistive service in healthcare (Yoon and Lee, 2018; Scoglio et al., 2019) and language and social behaviour learning in education (Van den Berghe et al., 2019). As robots ‘evolve’ into humans’ social and conversational partners, what does it mean for their interface design? How should a social robot’s appearance, voice and behaviours be designed to accommodate these changes?

The typical design of social robots incorporates anthropomorphisation, which means making a robot appear and behave like a human. For example, to add social presence and visual features such as eyes, ears or a mouth to a robot, project human-like faces or voices, or display emotions explicitly via facial expressions and speech. This design implies that human users can interact with a human-like robot socially (Bartneck et al., 2020, p.174). Many efforts have been devoted to developing human-like interactive technologies. For example, the market giant Microsoft developed more natural-sounding voices (Huang, 2018); Google used an AI system which can control its intonation by incorporating speech disfluencies (e.g., ‘hmm’s and ‘uh’s) and latency lengths when making phone calls to human users (Leviathan, 2018); the social robot company ‘Furhat’ offers a wide range of human-like customisable appearances, which can display various facial expressions (Furhat Robotics, 2023).

The technological developments offer progressively more options for human-like appearance, voice and behaviours. However, the user experience of spoken interaction has proved unsatisfactory (Moore et al., 2016). Although some studies show the more human-like an agent is, the more likeable it is (Brooks, 2002; Kühne et al., 2020), a causal relationship between a social robot’s anthropomorphic form and more natural social interaction cannot be shown (Kanda et al., 2004). The positive experience could be caused by other factors, such as the novelty effect or multimodal experience (Mayer and DaPra, 2012; Van den Berghe et al., 2019). In addition, human-like design has risks of falling into the ‘uncanny valley’ (Mori et al., 2012), which is proved mathematically by Moore (2012): the more human-like an object is, the more affinity it gains until a point where the eerie sensation occurs when the human-like objects highly yet imperfectly resemble actual human beings. There are also ethical concerns about deceptive human-like designs which generate fake sentimentality (Danaher, 2020; Hildt, 2021). Hence, a need arises to explore people’s perceptions of a social robot’s form in more depth.

Going beyond the first impression, what a social robot looks like, sounds like and behaves like would also affect people’s perceived action possibilities, namely, its ‘affordance’. The term ‘affordance’ was invented by ecological psychologist Gibson (Gibson, 1977) and has been brought into the psychological study of human-technology interactions by Norman. Simply put, affordance is a connection between what people see, what they think is possible and what they do with an object in a given situation (Norman and Love, 2004; Norman, 2008; Matei, 2020). People tend to assume that an agent’s affordance will be in accordance with its capabilities. In the case of human-robot interaction (HRI), if people encounter a human-like robot, they expect it to act like a human; if a robot can talk, people expect it to be able to hold a conversation in natural language (Bartneck et al., 2020, p.45). In the interaction, people may feel very disappointed and fall into the habitability gap when they find out that the robot cannot perform as it appears to be (Moore, 2017). Thus, it can be said that a robot’s affordances need to be appropriately designed to represent explicitly its capabilities and to shape users’ expectations of it (Huang and Moore, 2022).

### 2.2 The challenges of multi-modality and use cases

As embodied agents, social robots’ affordance is manifested in many ways, including their appearances, voices, and verbal and non-verbal behaviours. These multi-dimensional cues increase the risk of getting a certain aspect wrong and falling into the uncanny valley (Moore, 2012). The previous studies of ‘the Uncanny Valley’ mainly focus on robots’ appearances, voices and movements. Studies show that perceptual mismatches of facial features, such as artificial eyes on a fully human-like face, would cause negative affinity (MacDorman and Chattopadhyay, 2016); mismatched face-voice stimulus (Mitchell et al., 2011) could also reduce users’ sense of affinity. This study will extend the exploration of such alignment to a robot’s verbal and non-verbal behaviours in spoken HRI. In this study, we refer to a robot’s face and voice as its ‘static affordance’ because they are relatively stable during interactions. For example, a child-like robot would keep its child-like face and voice in interactions. Correspondingly, we refer to a robot’s verbal and non-verbal behaviours as ‘behavioural affordance’. For example, a robot can change its facial expressions, gazes, and verbal expressions to deliver messages in interactions. The question that interests us is how a robot’s static affordance should be aligned with its behavioural affordance. For example, how should a robot wearing a child-like look and voice behave in a conversation? What about a robot with a robot-like look and voice?

To investigate this question, we take into account what the robot is used for. Campa (2016) emphasised that scenario and persona are two essential aspects to ensure that the robot’s behaviours are as natural as possible. The study by Wilson and Moore (2017) about voices found that when robots, aliens, and cartoon characters’ voices are often changed to match the story, these voice alterations are connected to specific personae. Thus, when it comes to examining the alignment of affordances of a social robot, it is worth putting the robot in different use cases and investigating how the same static affordance design may be more appropriate for one robot role but less for another. For example, what kind of affordance is more appropriate for an informative use case in which a robot only provides information? What about an emotional use case in which a robot provides emotional values?

Finally, to capture people’s perceptions of social robots used in social situations, it is sensible to measure people’s social perceptions of them. Two dimensions widely used to measure people’s social perceptions are warmth and competence (Cuddy et al., 2008; He et al., 2019). The former captures the perceived friendliness and good intentions. The latter captures the perceived ability to deliver on those intentions. This study employs these two dimensions to measure what people expect robots to be like in different situational roles.

## 3 Research hypothesis and questions

Based on the above review, affordance design is crucial to social robots used in various social domains. Going beyond anthropomorphisation and the face-voice match, it is necessary to examine how behavioural affordance should be aligned with static affordance to help users form an appropriate and consistent perception of a social robot in given situations. Hence, this paper aims to understand further what matters in conversational HRI from three aspects: a robot’s static affordance (appearance and voice), behavioural affordance (behaviours) and situational roles. The research hypothesis of this paper suggests that a social robot designed with appropriate and consistent affordances helps users form more stable perceptions and gain more satisfactory experience in spoken HRI. The research questions (RQs) are as follows.

•RQ1: How does static affordance impact participants’ perceptions of a robot before and after interactions?
•RQ2: How does the use case impact participants’ perceptions of a robot before and after interactions?
•RQ3: How does behavioural affordance impact participants’ perceptions of a robot during interactions?

## 4 Research materials and methods

We designed an exploratory experiment that invited participants to complete a series of tasks. In this section, we firstly introduce the settings of two independent variables in the experiment: three static affordances and three behavioural affordances applied in conversational use cases. Examples of the dialogue transcripts of each use case are provided. Then, we explain the study design, focusing on the experiment procedure and measurements. Lastly, we give an overview of the participants’ recruitment and attendance, as well as data analysis plan.

### 4.1 Affordance and use cases settings

#### 4.1.1 Affordances

The robot used in this study is ‘Furhat’ (Furhat Robotics, 2023). It is a head-only robot with virtual faces that can be back-projected onto the semi-translucent masks. This feature makes using various face settings possible. ‘Furhat’ also has various human-like synthesised voices, including neutral British English. Additional voice effects can be generated to get a robot-like effect with the assistance of a plug-in voice changer *TC Helicon Perform VE*.

**FIGURE 1:**
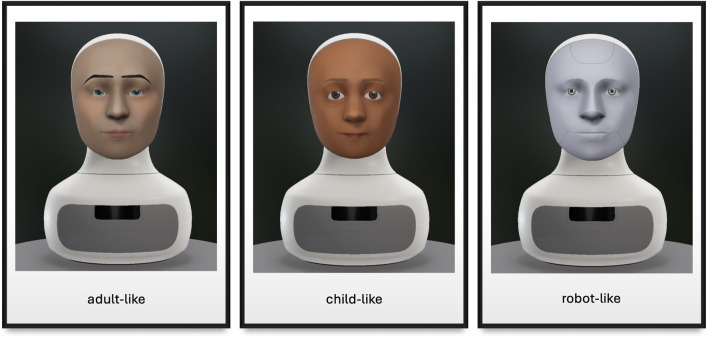
Three static affordance settings used in the experiment: adult-like, child-like and robot-like.

Three matched face-voice settings are used in the experiment. They are ‘adult-like’, ‘child-like’ and ‘robot-like’ affordances. Figure 1 shows what each setting looks like. The detailed specifics are as follows. The adult setting: mask = ‘adult’, face = ‘Alex’, voice = NeuralVoice.Brian. The child setting: mask = ‘child’, face = ‘Devan’, voice = NeuralVoice.Kevin. The robot setting: mask = ‘adult’, face = ‘Titan’, voice = NeuralVoice.Brian. The voice of the robot character is changed via the voice changer to make it sound like a *Dalek*, a robotic character in the British science fiction television programme *Doctor Who* (TC-Helicon, 2017, p.14).

#### 4.1.2 Conversational use cases

According to the transformation of the speech-enabled agents’ use cases as described by Moore (2017), social robots can be used in the physical, informative and emotional domains. These three are not mutually exclusive and can overlap in a dialogue scene. Since we focus on conversational interactions, this study does not consider the physical domain. We aim to study three conversational interactions: informative, emotional and hybrid.

In the experiment, the informative interaction is a question-and-answer interaction. In this use case, a robot plays the role of a respondent who encourages users to ask science and maths questions and provides answers. The emotional interaction is based around jokes. In this use case, a robot tells jokes and then responds to users’ non-verbal reactions. The hybrid interaction is a quiz scenario which combines informative and emotional elements. In this use case, a robot plays a quiz host who can ask multiple-choice questions, repeat questions or options, judge users’ answers, and comment on users’ responses and engagement.

These three use cases were programmed modular dialogue systems adapted from Furhat’s skill library (FurhatRobotics, 2017). The main adaptations of the dialogue include (1) changing the start and end status of the robot via the head and the face light settings. For example, the robot’s face will brighten up to indicate the robot is activated for a conversation. The robot’s face will be dimmed to indicate the end of a conversation. (2) adding Wizard-of-Oz (WoZ)^1^ operations to enable the experimenter to end the conversation and control the experiment time.

The robot can autonomously keep eye contact with participants by tracking their heads in social conversation. As for the emotional elements, facial expressions and verbal responses were programmed beforehand, which would either be triggered by users’ reactions or coupled with a robot’s response. The informative science role has limited facial expressions and uses limited sentimental words in the conversation. It shows limited affection. The joke role creates an amicable atmosphere by equipping the robot with functions to detect users’ smiles and then respond by smiling back. Its language contains positive and humble tones. The emotion range of the quiz role contains both positive and negative feelings. A summary table of emotional settings for each role is provided in Table 1.

**TABLE 1:** Emotion settings for three situational roles.

Robot	Facial expressions	Use of language
Informative Science Role	• Gaze away to indicate ‘thinking’ status	Limited use of sentimental words. Only comment on questions. E.g., ‘Good question’, ‘Great question’, ‘Interesting.’
	• Raise brow to indicate waiting for questions for a long time	
Emotional Joke Role	• smiling detection: Detect a user’s smiling and take smile back actions; calculate the number of milliseconds spent laughing	• Ask how users feel
	• 25% of the time, it returns a small smile	• React on users’ feelings. E.g., ‘I am sorry to hear that.’, ‘Great to hear.‘, ‘Wow! You really liked this joke!’
	• 50% of the time, it returns a big smile	• Offer to make people happy. E.g., ‘do something to cheer you up’, ‘increase your happiness a few notches’
	• After a smile, it waits 5 seconds	• Be humble. E.g., ‘I am trying to learn some humours’, ‘Could I test a few jokes on you?’
Hybrid Quiz Role	• Smile when answers are correct	• Short comment on right answers. E.g., ‘Great!’
	• Frown when answers are incorrect	• Short comment on wrong answers. E.g., ‘Too bad.’
	• Raise brows when asked to repeat a question	• Short comment on slow responses. E.g., ‘Too slow!’, ‘A bit too slow amigo.’
	• Surprise when asked to repeat options	• Give an order. E.g., ‘Look at me.’
	• Big smile when asking users to pay attention	

Tables of the dialogue flow and an example transcript of each use case are provided in Tables 2–4.

**TABLE 2:** Example dialogue transcript: science question and answer.

Dialogue stage	Robot	Users
Self-introduction	Hello! I am a science robot.	*Rate the first impressions*
Opening	Hi. I can answer questions about facts. I am by no means perfect, but I’m OK at science and mathematics. Don’t get too philosophical.	-
Invite questions	Go ahead and ask me about something.	What’s 5 plus 7?
Give answers	OK. *(Gaze away)* 5 plus 7 is 12.	-
Invite another question	Anything else?	How many planets are in the solar system?
Give answers	Let me think. *(Gaze away)* The answer is 8.	-
Invite another question	Do you have another question?	No. That’s it. Thank you.
End the conversation	Thank you for your questions. Goodbye for now.	-

**TABLE 3:** Example dialogue transcript: playing jokes.

Dialogue stage	Robot	Users
Self-introduction	Hello! I am a joke robot.	*Rate the first impressions*
Greetings	Hi there.	Hello.
Opening	I am wondering, are you happy today?	Yeah yeah, I am happy.
Invite users to test jokes	Great to hear. Then you are in the right mood! I am trying to learn some humour, you see. So, could I test a few jokes on you?	Yeah. OK. Go for it.
Tell a joke	Awesome. This one has been a success so far. What do robots do at lunchtime? *(Wait for 2 s)*	-
Give the punchline	Have a mega-byte.	*smile*
React to users’ responses	*(Smile back)* Yay, I am getting the hang of this.	-
Ask if to tell another	Would you like another one?	Yes.
Tell a joke	Sweet! This one has been a success so far. What happened when they shut down the robot motorway? *(Wait for 2 s)*	-
Give the punchline	Everyone had to take the R2 detour.	*smile*
React to users’ responses	*(Smile back)* Not bad, right?	-
Ask if to tell another	Would you like another one?	No, that’s OK.
End the conversation	Thank you for trying my jokes. Goodbye for now.	-

**TABLE 4:** Example dialogue transcript: playing quiz questions.

Dialogue stage	Robot	Users
Self-introduction	Hello! I am a quiz robot.	*Rate the first impressions*
Greetings	Hi there.	Hello.
Opening	Do you want to play?	Yeah, OK.
Start the game	I will ask you 5 multiple-choice questions. And we’ll see how many points you can get. Alright, here we go.	-
Ask a quiz question	What did the Wright Brothers invent in 1902? Car, airplane, motorbike or fighter jet?	Airplane.
Check the answer	*(smile)* That was correct. You now have a score of 1 After a smile, it waits	-
Ask another quiz question	What is the title of the famous novel by George Orwell? Of Mice and Men, The Great Gatsby, 1984 or The Lord of the Rings?	*silence*
Check users’ engagement	Too slow! Here comes the next question.	-
*continue questions and answers*	…	…
Calculate the score	That was the last question. You got 4 points.	
End the conversation	I hope you had some fun. Goodbye for now.	-

### 4.2 Study design

A mixed-method approach has been adopted to understand better the proposed research questions and gain insights from listening to participants’ feedback and comments. The study uses the convergent design, which collects quantitative and qualitative data at similar times (Creswell, 2014; Creswell and Clark, 2017). Integration of mixed methods occurs by explaining quantitative results with a qualitative approach and merging quantitative and qualitative results to form conclusions. (Fetters et al., 2013). In the study, the quantitative method is used to explore potential correlative relationships between independent variables (affordances and use cases) and people’s perceptions of the robot. For example, would different affordance settings affect people’s perceptions of a robot’s warmth and competence? The qualitative method is used to understand what factors make people feel differently.

The experiment included an online survey, a face-to-face interaction session and a post-experiment interview. The online survey collects participants’ background information, including their demographics, first languages and accents, and their experience of interacting with speech-enabled agents. The Technology-Specific Expectation Scale (TSES) (Alves-Oliveira et al., 2015) is used to collect and examine how pre-interaction attitudes. This is used as the baseline so we can compare how people’s perceptions change after interactions.

The in-lab interaction session requires participants to have three face-to-face spoken interactions with a social robot in an HRI lab. The 3*3 factorial design of the experiment creates nine conditions. Participants are assigned to complete interactions in three use cases, and the robot wears different affordance settings each time. The order of the interactions is semi-randomised, as shown in Table 5. Take three participants, for example. One participant interacted with an adult-like science robot, a child-like joke robot and a robot-like quiz robot; the next participant interacted with an adult-like joke robot, a child-like quiz robot and a robot-like science robot; the participant after that interacted with an adult-like quiz robot, a child-like science robot and a robot-like joke robot. This arrangement maximises the even distribution of each test condition to reduce the order effect.

**TABLE 5:** Example of ordering of experimental conditions.

Participants	Interaction 1	Interaction 2	Interaction 3
1	an adult-like science robot	a child-like joke robot	a robot-like quiz robot
2	an adult-like joke robot	a child-like quiz robot	a robot-like science robot
3	an adult-like quiz robot	a child-like science robot	a robot-like joke robot

A score sheet records participants’ first impressions of each robot and post-interaction ratings for each interaction. Considering the focus of the study is social attributes, we used the Robotic Social Attributes Scale (RoSAS) to measure people’s perception of a conversational role’s warmth and competence (Carpinella et al., 2017). We also used the Technology-Specific Satisfaction Scale (TSSS) (Alves-Oliveira et al., 2015) to examine participants’ perceptions of a robot’s capabilities.

To reduce the impact of the experimenter, the lab was divided into two parts with a black curtain, where the participant interacted with the robot in front of the curtain, and the experimenter sat behind the curtain. In the experiment, the experimenter controlled Furhat on a laptop. Operations included making a robot introduce its conversational role, starting the conversation and using WoZ buttons to end it.

**FIGURE 2:**
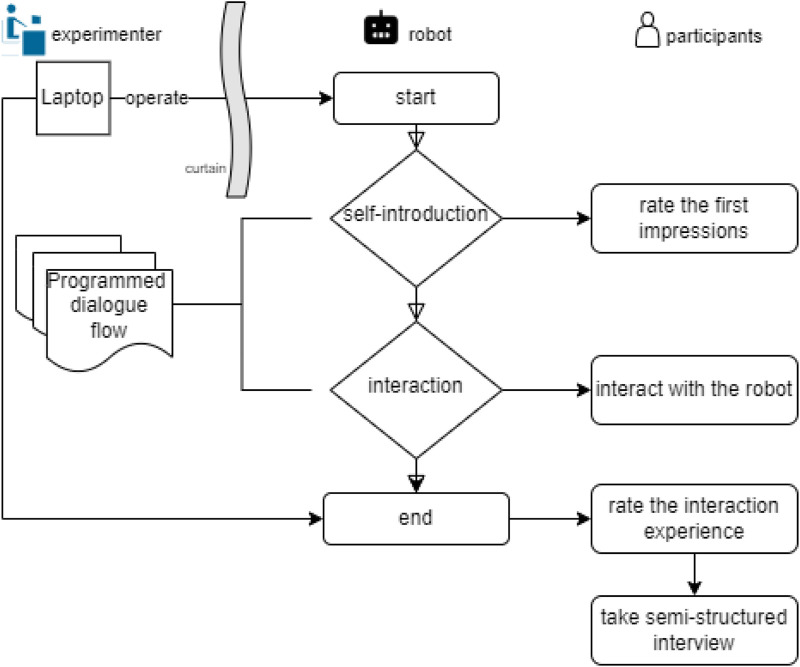
Diagram of the experiment flow of the in-lab session.

After the interaction session, participants took a post-experiment interview to provide more detailed comments based on their experience in the experiment. Four questions were used as a springboard to help participants recall different aspects of their interactions: (1) ‘How would you describe the robot’s language abilities?,’ (2) ‘How would you describe the robot’s interactive abilities?,’ (3) ‘How did you feel when you encountered difficult moments in the interaction?’ and (4) ‘How do you like the robot’s performance when encountering difficulties?’ Based on participants’ responses, the experimenter could also extend questions or ask for clarification. A diagram of experimental setup and flow is illustrated in Figure 2.

### 4.3 Participants, data collection and analysis plan

The experiment gained ethics approval from the University of Sheffield in September 2022 (Application Reference Number: 046753). It was launched on 25 November 2022 and lasted until 02 March 2023. Participants were recruited from the campus, including university staff and students. 70 participants completed the online survey. 52 of them attended the lab session, and their data were used in data analysis. Participants included females (n = 32), males (n = 18), and non-binary (n = 2) across different age groups with various backgrounds.

Age: Most participants are young adults, with 36.5% for 18–24 years old and 38.5% for 25–34 years old. Participants aged 35–44 years old, 45–54 years old, 55 and above are 11.5%, 7.7% and 5.8%, respectively.

Nationality and accent: Most participants are British (63.4%) and use English as their first language (72.8%)^2^. They report no accent, or their English accent rarely causes problems when speaking with others. Chinese participants make up 11% and report their English accent occasionally or often causes problems when speaking with others. Other nationalities take up 25.8%. Their first languages are Arabic, French, Indonesian, Spanish and Tamil. Some of them find their English accent occasionally causes problems when they speak with others (8.5%).

Experience with speech-enabled agents: 89.6% of all participants have experience with speech-enabled speakers, mostly for asking for a piece of information or giving it a command to do things. Most of the participants do not have any interactive experience with a social robot (52.3%).

156 samples were collected from 52 participants. Among them, 18 samples were collected for these three conditions: an adult-like quiz robot, a child-like joke robot and a robot-like science robot. Apart from that, the other six conditions contain 17 samples each.

The approach to analysis was twofold. The quantitative data are from the closed questions with a Likert scale collected via questionnaires. They were mainly analysed and visualised using IBM SPSS Statistics (Version 29.0.0.241). This offered an overview of the quantitative relationships between two independent variables (affordance settings and use cases) and the participants’ perceptions. It also allowed us to raise questions about observed changes. The second round of data analysis was qualitative. 52 semi-structured interviews were transcribed as 40,851 words. NVivo (Version 14.23.0) software is used in the iterative process of thematic analysis, with the main emphasis on factors contributing to people’s perceptions and providing potential explanations of raised questions in the quantitative analysis.

## 5 Results

### 5.1 Quantitative analysis

In the experiment, there are three static affordance settings on Furhat: adult-like, child-like and robot-like. Participants were required to give ratings using a Likert Scale from 1 to 5 for five items: likeability, trust, competence, warmth and motivation. These measurements were taken before and after interactions. The pre-interaction ratings were taken after the robot said, ‘Hello, I am a *(use case)* robot. The post-interaction ratings were taken after participants completed the conversation with the robot. The purpose of the quantitative analysis is to have an overall picture of participants’ perceptions and examine the effect of static affordances and use cases on people’s perceptions. Hence, we carried out the same analysis of the pre-interaction and post-interaction rating data, which included providing an overview of the data distribution on the five aspects via box plots, comparing means across all tested conditions, and examining the effect of two independent variables: static affordances and use cases. In addition, changes in data before and after the interaction help us identify further questions about ‘why’, which gives us more perspectives to analyse the interview data by means of abductive reasoning.

#### 5.1.1 Descriptive analysis

**FIGURE 3:**
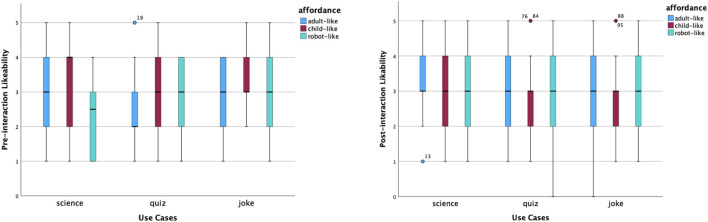
Distribution of likeability scores before and after interactions.

Figure 3 shows the distributions of the perceived likability ratings. Before interactions, the child-like science robot received the highest median score of likeability, whilst the adult-like quiz robot had the lowest. In both cases, many participants had similar views. The former was left-skewed. The latter was right-skewed. After interactions, all items shared the same median yet with different levels of agreement. Participants had somewhat more variability of the adult-like joke robot. Participants’ opinions on how much they liked a child-like quiz robot and science robots became more focused. Participants’ opinions regarding the robot-like joke robots remained the same, with the same median at a neutral level, the same variability and normal distribution. The child-like joke and quiz robots’ perceived likability had a couple of high outliers.

**FIGURE 4:**
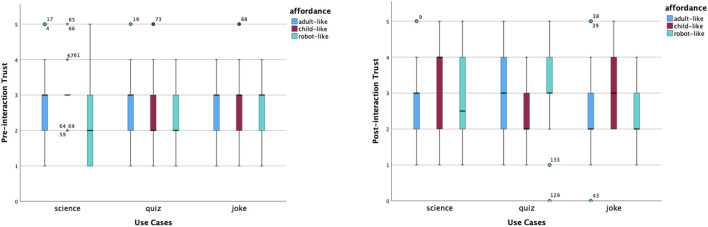
Distribution of trust scores before and after interactions.

Figure 4 shows the changes in the perceived trust. Before interactions, the median scores were the same across most conditions, apart from the robot-like science robot and the child-like and robot-like quiz robots. Distributions of perceived trust were relatively focused across most cases, apart from the robot-like science robot that had more variability. Participants’ ratings towards perceived trust of child-like and robot-like quiz robots were right-skewed. The child-like science robot had several far-out outliers. After interactions, the same individuals had more diverse opinions about how much they trusted robots in different scenarios and with different looks. The child-like science robot received the highest median score of perceived trust, with a left-skewed distribution. There was increased trust in both robot-like science and quiz robots.

**FIGURE 5:**
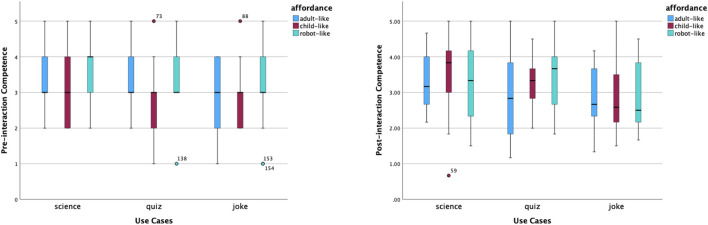
Distribution of competence scores before and after interactions.

**FIGURE 6:**
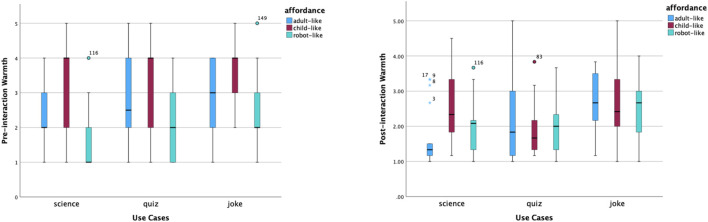
Distribution of warmth scores before and after interactions.

The shifted opinions were also reflected in perceived competence, warmth and motivation to interact. According to Figure 5, the distribution of perceived competence of robot-like roles was concentrated in the middle and high ranges. The robot-like science robot had the highest median of perceived competence. The distributions of perceived competence of the child-like quiz and joke roles were left-skewed. After interactions, opinions on the perceived competence of robot-like roles became more scattered, and opinions on the level of competence of child-like science and quiz roles increased relatively. In contrast, the perceived warmth of child-like roles was rated from high to low, as shown in Figure 6. In addition, the robot-like science role received the lowest median point before interactions. The median points of all robot-like roles were the same or higher after interactions. It is interesting to notice the very small variability of the perceived warmth of the adult-like science robot.

**FIGURE 7:**
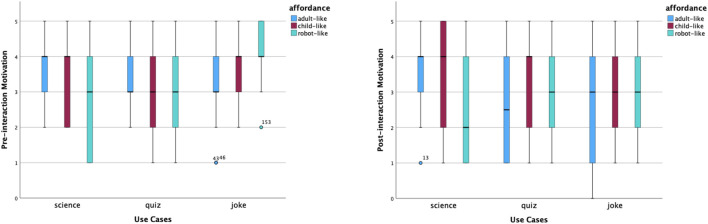
Distribution of motivation scores before and after interactions.

The motivation to interact is shown in Figure 7. Based on the median points before interactions, participants felt most motivated to talk to the adult-like science role, the child-like science and joke roles, and the robot-like joke roles. How much participants would like to talk to the robot-like science role had the widest variability. After interactions, participants would like to talk to an adult-like science robot again the most, according to the median point and the relatively narrower variability.

**FIGURE 8:**
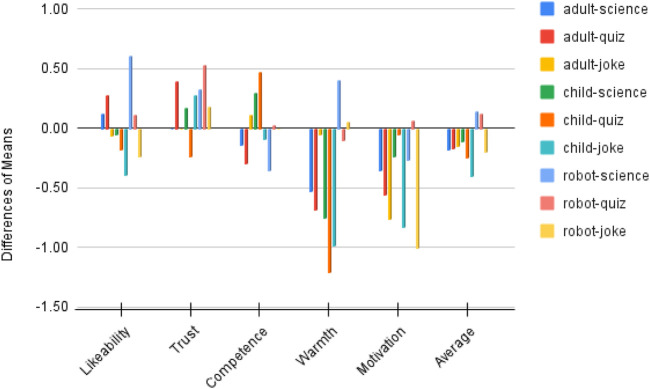
Changes of perceptions after interactions: bars going upwards above 0 mean the means of post-interaction ratings were higher than pre-interaction ratings; bars going downwards below 0 mean the means of post-interaction ratings were lower than pre-interaction ratings.

We also calculated the mean scores of each measured item under combinations of two variables, as shown in Table 6. To visualise the changes, we produced Figure 8. Overall, the average score of participants’ perceptions of robots decreased after interactions, apart from a robot-like science role and a robot-like quiz role. Specifically, before interactions, a child-like robot received the best first impressions in all three use cases, with *M* = 3.29 for the joke role, *M* = 3.28 for the science role and *M* = 2.95 for the quiz role. After interactions, child-like science and joke roles were still rated as the top one on average. However, among the three static affordance settings, participants’ perceptions of the child-like roles changed the most (−0.25 on average across use cases). This is mostly caused by the greatly declined ratings for ‘warmth’ and ‘motivation’ of child-like roles. In contrast to this, participants’ perceptions of the robot-like roles changed the least (0.02 on average across use cases). A robot-like science role gained higher post-interaction perception scores on likeability, trust and warmth.

**TABLE 6:** Means of perceptions before and after interaction.

Condition	Interaction	Likeability	Trust	Competence	Warmth	Motivation	Average
adult-science	pre-	3.00	2.76	3.47	2.24	3.76	3.05
	post-	3.12	2.76	3.33	1.71	3.41	2.87
adult-quiz	pre-	2.61	2.67	3.28	2.78	3.17	2.90
	post-	2.89	3.06	2.99	2.10	2.61	2.73
adult-joke	pre-	3.00	2.59	2.71	2.76	3.41	2.89
	post-	2.94	2.59	2.82	2.71	2.65	2.74
child-science	pre-	3.29	3.12	3.18	3.29	3.53	3.28
	post-	3.24	3.29	3.48	2.54	3.29	3.17
child-quiz	pre-	3.00	2.53	2.82	3.12	3.29	2.95
	post-	2.82	2.29	3.29	1.91	3.24	2.71
child-joke	pre-	3.33	2.78	2.94	3.67	3.72	3.29
	post-	2.94	3.06	2.85	2.69	2.89	2.89
robot-science	pre-	2.33	2.39	3.61	1.61	2.83	2.55
	post-	2.94	2.72	3.26	2.01	2.56	2.70
robot-quiz	pre-	2.71	2.53	3.35	2.12	2.88	2.72
	post-	2.82	3.06	3.38	2.02	2.94	2.84
robot-joke	pre-	3.18	2.47	3.00	2.47	4.12	3.05
	post-	2.94	2.65	3.00	2.52	3.12	2.85

#### 5.1.2 Correlational analysis

A two-way analysis of variance (ANOVA) was performed to determine if static affordances (adult-like, child-like, robot-like) and use cases (science, joke, quiz) had a significant effect on perceived likeability, trust, competence, warmth and motivation.

Perceived likeability: Before interactions, there was no statistically significant interaction effect (*p* = 0.44) between static affordances and use cases, no main effect of static affordances (*p* = 0.09) or use cases (*p* = 0.17). Regarding the post-interaction perceived likeability, no statistically significant interaction effect (*p* = 0.91) or main effects (*p* = 0.10 for the static affordances effect, *p* = 0.61 for the use case effect) were found.

Perceived trust: It was the same case for before and after interactions. For the pre-interaction perceived trust, the *p* values of the interaction effect, static affordances effect and the use case effect are *p* = 0.68, *p* = 0.23 and *p* = 0.64, respectively. For the post-interaction perceived trust, these values are *p* = 0.08, *p* = 0.93 and *p* = 0.77, respectively.

**FIGURE 9:**
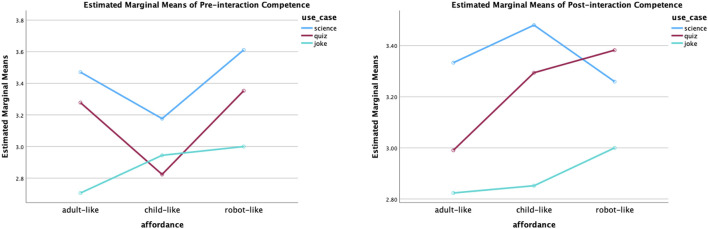
Two-way ANOVA plot of perceived competence. Statistically speaking, the science roles were significantly more competent in participants’ opinions before and after interactions.

Perceived competence: The analysis results of pre-interaction ratings revealed that use cases had a statistically significant main effect (*F* (2,147) = 4.17, *p* = 0.02). The effect size is small (partial eta squared = 0.054). Further, the post-doc test (Tukey’s test) for multiple comparisons found that science roles (*M* = 3.42, *SE* = 0.94) were rated significantly (*p* = 0.01) more competent than joke roles (*M* = 2.88, *SE* = 0.98). However, there was no significant interaction effect of static affordances and use cases or significant main effect of the static affordances. In addition, the statistically significant impact of the use cases was also reflected in the post-interaction ratings of the perceived competence (*F* (2,147) = 2.97, *p* = 0.05). The effect size remains small (partial eta squared = 0.04). The post-doc test (Tukey’s test) for multiple comparisons found the same result, that is, the science roles (*M* = 3.36, *SE* = 1.02) were considered more competent than the joke roles (*M* = 2.89, *SE* = 0.92); the difference was statistically significant (*p* = 0.05). The plot of the two-way ANOVA of perceived competence is shown in Figure 9.

Perceived warmth: Before interactions, static affordances (*F* (2,147) = 16.75, *p* < 0.001) and use cases (*F* (2,147) = 3.42, *p* = 0.04) both had a statistically significant effect. The effect sizes are small (partial eta squared = 0.19 for the static affordance, partial eta squared = 0.04 for the use cases). According to the post-hoc test results, the child-like robots were considered statically significantly warmer than the adult-like robots with *p* = 0.002; the adult-like robots were considered statically significantly warmer than the robot-like robots, with *p* = 0.05; the child-like robots were considered statically significantly warmer than the robot-like robots with *p* < 0.001. The means and standard deviation of each affordance setting are ordered as follows: the warmest child-like robots (*M* = 3.37, *SE* = 1.24), the warmer adult-like robots (*M* = 2.60, *SE* = 1.14) and the least warm robot-like robot (*M* = 2.06, *SE* = 1.09). In addition, the joke roles (*M* = 2.98, *SE* = 1.18) were rated warmer than the science roles (*M* = 2.37, *SE* = 1.34). The difference was statistically significant (*p* = 0.02). There was no statistically significant interaction effect between static affordances and use cases.

**FIGURE 10:**
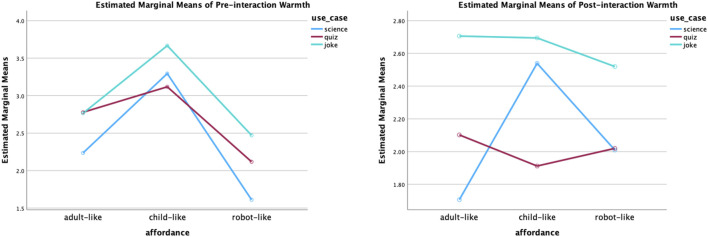
Two-way ANOVA plot of perceived warmth. Before interactions, both static affordance (child-like adult-like robot-like) and use case (joke science) played statistically significant roles in perceived warmth. After interactions, statistical significance was found between joke and science roles, as well as joke and quiz roles.

After interactions, the static affordance was not identified as the main effect of the perceived warmth. The use case, on the other hand, maintained its statistically significant effect (*F* (2, 147) = 7.34, *p* < 0.001). The effect size is small (partial eta squared = 0.09). Further, the post-hoc test found two statistically significant differences. The joke roles (*M* = 2.64, *SE* = 0.92) were considered warmer than the science roles (*M* = 2.08, *SE* = 0.93) and the quiz roles (*M* = 2.01, *SE* = 0.91), with *p* = 0.006 and *p* = 0.002, respectively. The plot of the two-way ANOVA of perceived warmth is shown in Figure 10.

Motivations: Before interactions, the use case was found to have the main statistically significant effect (*F* (2,147) = 4.00, *p* = 0.02). The effect size is small (partial eta squared = 0.05). The post-hoc test shows that participants were more motivated to interact with the joke roles (*M* = 3.75, *p* = 1.05) than the quiz roles (*M* = 3.12, *p* = 1.20). The difference is statistically significant (*p* = 0.02). After interactions, there was no statistically significant interaction effect (*p* = 0.29) or main effect of static affordance (*p* = 0.57) or use case (*p* = 0.75).

To further understand participants’ satisfaction with a robot’s performance, especially their perception of a robot’s competence, we examined the Technology-Specific Expectation Scale (TSES) and the Technology-Specific Satisfaction Scale (TSSS). Both scales contain two dimensions: fictional views and capabilities. Each dimension contains 5 items. The results in Table 7 show that the fictional views that people hold about robots were dispelled, especially the robot’s ability to perceive what participants would do before they do it (−43.7%) and its ability to understand their emotions (−30.9%). As for the perceived capabilities, participants felt more positive about the robot’s ability to recognise their gaze movements and to understand them, with 4% and 11.4% increases in rating, respectively. However, their confidence in interacting with the robot decreased by 10.5%.

**TABLE 7:** Pre-interaction expectations of robots and post-interaction satisfaction level, shown on two dimensions: fictional views and capabilities, and five items under each dimension.

	Before interactions—TSES (mean)	After interactions—TSSS (mean)	Differences (%)
**Fictional Views Dimension**	**2.6**	**1.7**	**−18.0**
The robot has superhuman capacities	3.17	1.63	−30.9
The robot is more than a machine	2.15	1.60	−11.2
The robot is able to perceive what I am going to do before I do it	3.62	1.43	−43.7
The robot is similar to the robots I see in movies	2.58	2.60	+0.5
The robot is able to read my thoughts	1.52	1.33	−3.7
**Capabilities Dimension**	**2.7**	**2.5**	**−4.0**
I am able to interact with the robot	3.65	3.13	−10.5
The robot can understand my emotions	2.73	1.86	−17.4
The robot is able to recognise when I look at it or when I shift my gaze to something else	2.56	2.76	+4.0
The robot has a sense of humour	2.29	2.05	−4.7
The robot is able to understand me	2.10	2.67	+11.4

We performed two-way ANOVA to determine the effect of static affordances and use cases on perceived capabilities. For the average score, it was found that the assumption of homogeneity of variance was violated when we ran Levene’s Test for Equality of Variances: the *p* values were greater than 0.05. Based on this, we raised the significance threshold from *p* = 0.05 to *p* = 0.01. There was no statistically significant interaction effect or main effect of either independent variable by applying this threshold (*p* = 0.54 for interaction effect, *p* = 0.89 for static affordance, *p* = 0.03 for use cases).

Further, we examined each item related to the perceived capabilities. As shown in Table 8, there was no statistically significant interaction effect or main effect of either independent variable in most cases, apart from the following two cases. Statistically speaking, the use case had a significant effect on perceived capabilities to understand emotions and have a sense of humour. The joke roles (*M* = 2.37, *SE* = 1.43) were considered more capable of understanding participants’ emotions than the science roles (*M* = 1.62, *SE* = 1.05) and the quiz roles (*M* = 1.60, *SE* = 1.07). The *p* values were 0.005 and 0.004, respectively. Participants also considered the joke roles (*M* = 2.79, *SE* = 1.39) to have more sense of humour than the science roles (*M* = 1.50, *SE* = 0.78) and the quiz roles (*M* = 1.87, *SE* = 1.34), with *p* < 0.001 for both differences.

**TABLE 8:** Two-way ANOVA result of perceived capabilities in TSSS rating. The use case was identified as the main effect for perceived capabilities to understand emotions and have a sense of humour.

Item	Interaction effect	Main effect - affordance	Main effect - use case
C1 I am able to interact with the robot	*p* = 0.83	*p* = 0.89	*p* = 0.17
C2 The robot can understand my emotions	*p* = 0.60	*p* = 0.97	*p* = 0.001, *F* (2.147) = 6.82, partial eta squared = 0.09
C3 The robot is able to recognise my gaze	*p* = 0.64	*p* = 0.74	*p* = 0.22
C4 The robot has a sense of humour	*p* = 0.11	*p* = 0.43	*p* = < 0.001, *F* (2,147) = 16.14, partial eta squared = 0.18
C5 The robot is able to understand me	*p* = 0.56	*p* = 0.99	*p* = 0.19

#### 5.1.3 Summary of quantitative analysis results

These findings suggest that, overall, participants’ perceptions of a robot varied across all three static affordances (adult-like, child-like and robot-like) and all three use cases (informative, emotional and hybrid). From the perspective of static affordances (face and voice), a child-like affordance won the best first impressions by a statistically significant margin due to its highly perceived warmth. However, such perception dropped greatly in interactions. This makes a child-like face and voice the affordance with the greatest perceptive change from pre-interaction to post-interaction. The affordance effect on perceived warmth was also reflected in robot-like roles. As opposed to child-like roles, robot-like roles were perceived as the least warm ones, especially the robot-science role. However, robot-like roles had the smallest perception gaps. Actually, robot-like science and quiz roles were the only two roles that had positive perception changes (on average).

As discussed above, the static affordance’s effect is related to pre-interaction warmth. The use case factor, including behavioural affordances during interactions, has a statistically significant effect not only on perceived competence, warmth and motivation to interact before interactions but also on perceived warmth in interactions. An informative use case, such as the science robot in this study, was perceived as more competent and less warm. On the opposite side, an emotional role, such as the joke robot in this study, was perceived as less competent and warmer. Such statistically significant differences remained the same before and after interactions. Additionally, the behavioural affordances associated with the emotional joke role increased participants’ perception of emotion-related capabilities, such as perceiving emotions and having a sense of humour.

Questions that need to be investigated further include what factors contributed to the drop in perceived warmth, especially for child-like robots, and what factors contributed to the small perception gaps of a robot-like robot. Additionally, it is interesting to note that participants did not feel confident interacting with robots after interactions. These questions will be discussed in the abductive reasoning part of the qualitative analysis.

### 5.2 Qualitative analysis

**FIGURE 11:**
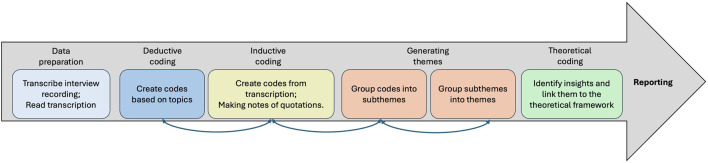
The process of qualitative analysis.

The process of qualitative analysis is shown in Figure 11. Both deductive and inductive methods are used in the coding process to allow the data to speak so the experimenter can identify topics, patterns, and themes that are connected to research questions in the theoretical framework (Vanover et al., 2021). The coding process is an iterative process. The presentation of findings will start with summative statements about what worked and what did not for each affordance aspect, including static affordances (face, voice) and behavioural affordances (facial expressions and language behaviours). Then, the analysis moves on to the emerging themes to discuss how affordances affect people’s perceptions of a robot in given use cases. Lastly, to answer questions raised from the quantitative analysis and unexpected replies in the interview, abductive reasoning is used to draw potential answers from the interview content. For example, what makes a child-like robot so likeable and what makes its perceived warmth drop significantly? Why do participants feel awkward when talking with a robot?

#### 5.2.1 Summative results: What works and what not

Generally, participants found the embodied face highly stimulating to look at. Facial expressions and eye movements made the robot more present, like someone who can think and interact. Participants reported that these movements of a robot, such as moving its head to keep eye contact, blinking and raising eyebrows, delivered a sense of engagement. Participants felt they were listened to and, therefore, had a sense of engagement. They even had a sense of connection when a robot returned a smile in response to their smiles and laughed at the right time. In this way, interacting with such a robot seems more natural. A few comments mentioned that a robot’s eye movement was bad or the smile was more like a ‘smirk’. But they thought it was better than nothing.

This sense of engagement was weakened when participants found the facial expressions repetitive. In other words, the movement was not based on a user’s input and did not reflect perceptions of users’ input. In this case, participants felt that an embodied face did not really contribute to interactions with a robot. Another type of social failure was that participants perceived unwanted mocking expressions or too much attention via eye contact.

“I enjoyed the way that (it looked at me). Yes, it felt like it was paying attention to me, but it could be a little much[…]Yeah, so that felt a little unnatural.”

“Really good at eye contact, almost to an uncomfortable level where it felt like I was in a staring contest.”

In addition to social failures, participants also pointed out technical failures. They include miscalibration of eye contact or failing to track users’ head movement. The design issues include off-putting robotic eyes, weird eye-moving tracks and flawless skin (mask) that makes facial expressions less easy to interpret. A few participants did not notice the function of head movement, eye movement or facial expressions at all. As explained by participants, it was partly because of their interaction habits in human-human interaction, partly because they did not move in the experimental interactions. When participants happened to be naive users of the social robot, they waved to the robot and found that the robot’s head movement only tracked their heads, or their hands, which was confusing.

Specifically, what did participants comment on various affordances and use cases?

The robot-like face was perceived as completely machine-like and less emotional. This perception made participants feel that the robot-like robot fit more naturally in a science role. Its robot-like voice was not popular. When it came to telling jokes and saying slang words such as ‘sweet’ and ‘awesome’, participants found it unexpected and funny.

In contrast, a child-like robot looked younger and cuter to participants. A few participants found it not amicable when it came to playing a science role. Participants also did not expect to receive judging comments from a child-like robot. ‘I do not like when the kids tell me it is too bad.’, one participant said. A couple of participants mentioned that the child-like face and voice did not match well, with a more child-like sound but less child-like look. There was little comment on an adult-like robot.

As for use cases, participants found quizzes and jokes more engaging. The quiz robot was found to be more interactive, competent and sociable. Participants noticed its richer range of facial expressions. They also found it was easy to interact with a quiz robot because it was straightforward. It would be more likeable if it could provide answers. The joke robot’s emotion detection and reaction function was well-received. Participants considered it a more human-like manner. Although the interpretation is not always right or clear, participants felt the robot is more interactive.

The science robot, unfortunately, seemed to bring ‘the most stressful’ interaction. Participants complained about the mismatch between the role it claimed to play and its true capabilities. ‘It told me it was a science robot. And it could not answer a lot of questions.’ one participant said. This mismatch increased uncertainty in interactions. Apart from that, the lack of emotion within the scenario made participants feel the science robot did not care.

#### 5.2.2 Emerging themes

Manner Matters More: Participants seemed to enjoy the interactions more when they felt engaged and connected. Rather than a human-like look or voice, the sense of engagement came more from human-like interactive manners. For example, pay attention and show interest. This comes from active listening and responsiveness. The summative results in the previous sub-session show that the robot used in this study appears to be listening. But its responses sometimes show a disconnection between what it listens to and how it reacts. Thus, it causes decreased motivation for interactions, as quoted below.

It was listening but not interacting.

Interactive manners were also reflected in taking and reacting to participants’ input and producing variable responses. Otherwise, the interaction seemed more like a programmed process. The repetition of expressions also enhanced this feeling. The repetitive emotional expressions, such as ‘Better luck next time, amigo!’, could be considered as ‘not genuine’.

The one with the quiz one…that did not seem that interactive because it was just the robot talking to me, and it was not taking much input from me. The child was…So, it had everything to be like a human being, let’s say. But it did not feel like a human being because the language was quite repetitive.

The interactive manners also connect to a robot’s appearance, voice and behaviours. For example, participants perceived a better match between a robot-like look and a science role. When there were mismatches between what a robot looked and sounded like and its behaviours, participants had different reactions. They found a robot-like robot telling jokes funny but found a child-like robot saying ‘too bad’ rude.

User Feelings of Uncertainty: One of the main feelings around interacting with robots revealed by the interviews was uncertainty. For example, there were a lot of comments reflecting frustration because of the lack of clarity on the robot’s competence. This could be caused by the mismatch between the situational role and its true capabilities, as demonstrated by the science robot.

I did not know what to ask it or what it might know. And so it sort of limited what I felt I could talk to it about…I did ask the 5 plus 5, but then I felt a bit stupid because I was like, obviously know what that is.

For those who cannot come up with any questions to ask, the setting puts them under pressure. It worsened when participants felt *‘completely blank about anything to do with science or maths’* (quoted). From this perspective, the quiz robot did better by leading a structured conversation. Participants then felt more relaxed as followers in interactions.

Another uncertainty created by the lack of clue for the cause of interaction failures or clues for how to move on. For example, the robot kept saying ‘I did not get it’ without providing more useful information. This made participants wonder: ‘Did it just fail to look up the information, or did it not understand what I was asking?’ After a few attempts to repair but not succeed, participants felt powerless: *‘*There was nothing I could say there.’ These uncertainties put forward the challenge of balancing free-style speech within the robot’s capabilities. As one comment puts it, the experimental robots only work ‘if you know that’s what you’re doing.’.

#### 5.2.3 Abductive reasoning

##### 5.2.3.1 What makes a child-like robot less likeable after interactions?

In the quantitative analysis part, it was found that a child-like robot was perceived to be less likeable and warm after interactions. Why so? The qualitative analysis confirmed that the static affordance design of the child-like robot was well received. *‘Friendly’* is the keyword associated with its face and voice. However, this means the alignment of multimodal cues for a child-like robot would be expected to be higher. For example,

The voice did not change; it had no inflection. (It) made the use of slang or like specific words a bit awkward […] It was a kid saying ‘sweet’, and, uh, usually when people say ‘sweet’, like, the voice goes higher, like, an exclamation. But it stayed the same kind of…

In addition, it was found that people had particular expectations for the child-like robot’s facial expressions and language behaviours, such as a relatively high level of expressiveness, enthusiasm, and politeness. So, when a child-like robot plays an informative science role which does not have many emotional behaviours, the mismatch could cause disappointment; when a child robot says ‘too bad’ if a participant misses a quiz question, it makes participants feel like they are being judged by a child. When such mismatches between the expectations and actions happen, the originally perceived friendliness cannot last and change to a new perception: ‘not amicable’.

##### 5.2.3.2 Why did participants have less confidence in having a conversation with a robot after interactions?

It may be natural to blame the conversation’s failure on a robot’s technological limits. For example, it cannot accurately recognise people’s speech or let participants talk over it. However, that is not the full picture. In fact, participants provided more specific words for their ‘less confident’ status: ‘awkward’ and ‘powerless’.

One explanation for the awkwardness might be that the robot was perceived as a social actor instead of a machine. Thus, making mistakes in front of a social actor was considered as a social failure that makes them feel awkward.

When it is just me looking at a screen, there’s not a face and a voice looking back at me, so if I do something wrong, I think, oh well, nobody saw that. Okay. But with the voice and the face, actually, it makes it slightly feel more like a social interaction, if that makes sense.

Another explanation lies in the robot’s capabilities to collaborate. Most participants did not feel that the robot recognised it when things went wrong. It ‘just moved on’. There were some strategies in the experimental use cases, which can be categorised as follows. Type 1: asking users to repeat or use specific expressions (e.g., ‘yes/no’). Type 2: indicating users to do something different in an implicit way, either by waiting in silence or repeating ‘I did not get it.’.

From the participants’ perspective, Type 1 is more explicit. It shows the robot’s limits, yet it is clear how to move on. Type 2 is too implicit and lacks clarity, which leaves room for participants’ own interpretations. It could be risky for interlocutors who cannot identify shared knowledge and experience. Participants tried rationalising a robot’s failures based on their understanding or experience with other speaking agents. They raised their voices, spoke slower, used simpler words or changed topics. But there is no more guarantee for success than when we try to communicate with people who do not speak our own language. While some participants took a robot as a developing tool and became more understanding, others felt very frustrated and annoyed.

Explicit strategies may work better. This also carries potential issues. When the robot asked participants to choose between ‘yes or no’ after it failed to understand their replies in the first place, there were a lot of negative comments. It was helpful but ‘too formal’, ‘not interacting’ and even ‘demanding’ and ‘threatening’. For more self-reflective participants, this expression made them feel they did something wrong and feel anxious.

What made things worse was the robot’s repetition after the participants’ repair efforts did not work. For example, after participants hear ‘I’m sorry, I did not understand you, can you say that again?’ a few times, they would know it did not understand them. Participants felt there was no need to hear the full expression again. The repetitive signal indicating not understanding did not provide further help but built up frustration. Participants feel ‘It does not care.’

These comments juxtaposed real and perceived evidence to show the invisible need for collaboration in a conversation. As human interlocutors, participants felt they had to put more effort into such collaboration without enough clues. Participants would like to see the robot’s engagement through its connection, listening status and reactions, which should correspond with what they actually say or do. For instance, a face with patterned expressions is less likely to be considered interactive. In addition, it shows the need to reduce cognitive barriers. For example, the robot needs to have a clear capabilities boundary to reduce participants’ effort to explore; it needs to adjust its behaviours so as not to make individuals uncomfortable. The failure of collaboration makes the robot ‘just be there’ instead of actually ‘being there’.

## 6 Findings and discussion

We separate this discussion into three parts: exploratory analysis of the effects of affordances and use cases on participants’ perceptions, implications and limits.

### 6.1 Effects of affordances and use cases on perceptions

From the overview of pre-interaction data distribution, it can be said that people have a preconceived stereotype of different looks and voices. For example, child-like robots are not so competent but friendly; a robot with a machine-like face and voice is more capable but not very friendly. Statistically speaking, the static affordances of a robot (face and voice) significantly affect people’s perception of how warm a robot appears to be at first glance across all static affordance settings (adult-like, child-like and robot-like), but they do not affect perceived likability, trust, competence or motivation to engage with the robot.

A child-like robot was considered friendly and warm, which made a very good first impression. The popularity of a child-like robot coincides with the positive perception and attitude towards the young-looking face (Bartneck et al., 2020, p.181). However, such a good impression could be a double-edged sword. This is because what a robot looks and sounds like shapes people’s expectations of their behaviours, such as how expressive or polite they need to be. These expectations then affect people’s perceptions of how fit a robot is for the given roles. In the case of a child-like robot, high demands are placed on aligning its behavioural affordance with its childlike look and voice, such as its tone of voice, and how expressive and enthusiastic it needs to appear and the choice of words when giving comments. If the child-like robot’s behaviours are not up to such expectations, it would create mismatched perceptual cues. It then causes the gap, which is similar to the ‘uncanny valley’. Thus, the findings of perceptions of a child-like robot in this study confirm the importance of aligning multimodal cues for social robots. As opposed to the previous study, which shows users would have lower expectations and more tolerance toward an infant-like robot, the current study shows that the perceived warmth declined greatly when a child-like robot’s behaviours did not match its perceived warmth at first glance.

The robot-like look and voice create an opposite impression. Its artificial, machine-like look was not highly regarded. It started with lower expectations and progressively gained more likability, trust, and warmth via its human-like manner, such as showing attention and interest by gazing and smiling back functions. It gained the smallest gaps between first impressions and post-interaction perceptions.

In comparison with face and voice, the use case factor matters more in the way that they significantly affect people’s perception of a robot’s competence and warmth before and after interactions. An informative use case, such as a science role, is statistically expected to be more competent than an emotional role, such as a joke role. The other way around, the emotional joke role is expected to be warmer than the informative science role. In the study, people are statistically more motivated to interact with a joke role than a quiz role. Here, we would like to clarify the relationship between use cases and behavioural affordances again in this study. The use case is just a term, such as ‘science’, ‘quiz’ or ‘joke’; behavioural affordances are more related to actions. Before the interaction begins, a use case’s impact on perception may be influenced by stereotypes related to the term; after the interaction, the impact on perceptions is more in terms of behavioural affordances. Based on the results of the analysis, it is safe to say that behavioural affordances matter more to manage the perception gaps.

According to the qualitative analysis, there are two types of behavioural affordances. One type is role-specific, which is associated with a robot’s static affordance. One good example is the joke robot, which has a certain capability to recognise users’ emotions and some sense of humour. Another good example is the child-like robot in this study, as explained above. The other type is generic, which can be applied to all social robots. It includes interactive and social behaviours that make people feel more engaged, such as keeping eye contact and smiling back. More importantly, these behaviours need to be responsive to users’ input with varieties. A robot’s responses need to be meaningful. This echoes the ‘Cooperative Principle’ proposed by Grice (1975). These principles describe what meaningful information should be like, which are maxims of quantity, quality, relation and manner. The maxim of quantity: informative but not too much. The message sender needs to deliver the information in a way that the receiver can understand and not get overwhelmed. This is based on the sender’s assumption of what the receiver may already know. From this perspective, meaningful messages are not only the ones that bring new information but also those that help to reduce the other party’s cognitive uncertainty or burden in the conversation. The concerns raised in the qualitative analysis show that a robot needs to be more informative when indicating its failure in interactions. Additionally, this requires signals to be true, as stated in the maxim of quality, not just perform repetitive programmed behaviours; be relevant according to the maxim of relation in the sense of building common ground and coordinating communication efforts; also be clear and organised according to the maxim of manner to reduce uncertainty.

Further, there is another question to answer: is there a direct link to indicate which affordance fits better with which type of use cases? Unfortunately, no direct link is identified. Given that static affordance and use case both have statistically significant effects on perceived warmth, it is natural to think a robot with warm static affordances would match a use case that requires warmth. Actually, the child-like joke robot was rated highest before interactions. But it was the bottom one among three child-like roles after interactions. If its behavioural affordances could fit better, it may be another story. Interestingly, the robot-like robot, which was considered not so warm, had a joke role that was better perceived than its quiz and science roles. Mismatches may not always cause trouble. Some cause decreased perceptions (e.g., a child-like robot behaves rudely). Some mismatches may be unexpectedly funny (e.g., a robot-like robot saying ‘sweet’).

### 6.2 Implication

This exploratory study identifies the effect of affordances and use cases on people’s perceptions of a robot. Simply put, if one is looking for a robot that leaves a good first impression, a child-like robot is better than an adult-like robot or a robot-like robot. If one cares more about narrowing the perception gap, a robot-like robot would perform well. Additionally, it highlights the importance of behavioural affordances, including designing generic behaviours to keep users engaged and aligning the role-specific behavioural affordance with a robot’s static affordance. What’s more, the design and use of social robots require a further understanding of use cases, which play a statistically significant role in determining the need for competence and warmth. These findings could be applied to the affordance design field and used by social robot designers, engineers and users.

### 6.3 Limitation

The shortcomings of this study include the following aspects. The study was performed on a specific robot (‘Furhat’) with three affordance settings and three use cases. So, extending it to different sorts of robotic settings would be useful to validate the findings. The adult-like, child-like and robot-like settings were chosen by the experimenter. It would benefit from double-checking participants’ perceptions. The data collection was performed with the robot present closely to the participants. This might have inhibited the expression of negative comments. The pre-interaction ratings of ‘warmth’ and ‘competence’ were collected by two questions. The post-interaction ratings of these two items were collected via the RoSAS. This weakened the reliability of changes between pre- and post-interaction perceptions. In addition, the study was designed and started before the launch of ChatGPT. In the later stage of the experiment, participants may have experience of interacting with ChatGPT. This may affect their expectations and experience of social robots used in this study. What’s more, participants came to the experiment as volunteers, not for personal use. Hence, their motivations to interact with robots may differ from those of real-life users. Most of the participants were from a university campus. It would be useful to test finding with participants from a more diverse background. Further, the experimenter’s personal interests and knowledge of social robots may steer the coding of the qualitative data and theme generation.

## 7 Conclusion and future works

What a robot should look, sound and behave like is essential to designing an effective conversational social robot. In this study, we examine how static affordances (face and voice) and behavioural affordances (verbal and non-verbal behaviours) affect people’s perceptions of a robot in informative, emotional and hybrid use cases (science, joke and quiz roles). The results show that static affordances have a statistically significant effect on people’s first impressions of a robot’s warmth. A child-like robot would be perceived as the warmest role. Comparatively, a robot-like robot would have the lowest perception of warmth. It is also found that use cases have a statistically significant effect on people’s first impressions of a robot’s competence, warmth and their motivation to interact, as well as post-interaction perceived competence and warmth. An informative role would be considered more competent yet less warm than an emotional role. In addition, it shows the importance of aligning static affordance with behavioural affordance to avoid a big drop in perceived warmth. In general, behavioural affordances should be genuinely responsive to users’ input and provide more information to collaborate with users in interactions. These findings prove that freedom comes at a cost. Taking off-the-shelf technologies and assembling a social robot with whom to communicate could lead to ineffectual HRI. A social robot with appropriate and consistent affordances built into its design should be seen as fundamental to effective communication and usability.

The directions to explore in future include many potentials. For example, how to further understand types of use cases from the perspectives of warmth and competence, how to design behavioural affordance to improve a robot’s abilities to collaborate when things go wrong, how to personalise affordance design by taking into count users’ perspective and why some mismatches between a robot role and its use case are more tolerated than other. Going beyond human-like appearance and voice, it is important to adopt behavioural characteristics that are appropriate to their physical makeup and cognitive capabilities.

## Data Availability

The datasets presented in this article are not readily available because they are part of an ongoing project which aims to be completed by the end of 2024. Requests to access the datasets should be directed to the corresponding author.

## Footnotes

1 The WoZ method means the researcher acts like an unseen ‘wizard’ who operates the robot remotely with a computer system in a human-robot interaction experiment. In this way, the participants believe a robot to be autonomous.

2 If participants hold dual nationalities and one of them is British, they are categorised as “British” here. If participants consider more than one language as their first language (L1), and one of the languages is English, they are categorised as L1-English in this study.
